# Effective endothelial cell and human pluripotent stem cell interactions generate functional insulin-producing beta cells

**DOI:** 10.1007/s00125-016-4078-1

**Published:** 2016-08-27

**Authors:** Dodanim Talavera-Adame, Orison O. Woolcott, Joseph Ignatius-Irudayam, Vaithilingaraja Arumugaswami, David H. Geller, Donald C. Dafoe

**Affiliations:** 1 Comprehensive Transplant Center, Department of Surgery, Cedars-Sinai Medical Center, 8900 Beverly Boulevard, 251E, Los Angeles, CA 90048 USA; 2 Board of Governors Regenerative Medicine Institute, Cedars-Sinai Medical Center, Los Angeles, CA USA; 3 Diabetes and Obesity Research Institute, Cedars-Sinai Medical Center, Los Angeles, CA USA; 4 Pediatric Endocrinology, Children’s Hospital, Los Angeles, CA USA

**Keywords:** Endothelium, Insulin secretion in vitro, Stem cells

## Abstract

**Aims/hypothesis:**

Endothelial cells (ECs) play an essential role in pancreatic organogenesis. We hypothesise that effective in vitro interactions between human microvascular endothelial cells (HMECs) and human pluripotent stem cells (hPSCs) results in the generation of functional pancreatic beta cells.

**Methods:**

Embryoid bodies (EBs) derived from hPSCs were cultured alone (controls) or with ECs in collagen gels. Subsequently, cells were analysed for pancreatic beta cell markers, and then isolated and expanded. Insulin secretion in response to glucose was evaluated in vitro by static and dynamic (perifusion) assays, and in vivo by EB transplantation into immunodeficient mice.

**Results:**

Co-cultured EBs had a higher expression of mature beta cells markers and enhanced insulin secretion in vitro, compared with controls. In mice, transplanted EBs had higher levels of human C-peptide secretion with a significant reduction in hyperglycaemia after the selective destruction of native pancreatic beta cells. In addition, there was significant in vitro upregulation of bone morphogenetic proteins 2 and 4 (BMP-2, 4) in co-cultured cells, compared with controls.

**Conclusions/interpretation:**

ECs provide essential signalling in vitro, such as activation of the BMP pathway, for derivation of functional insulin-producing beta cells from hPSCs.

**Electronic supplementary material:**

The online version of this article (doi:10.1007/s00125-016-4078-1) contains peer-reviewed but unedited supplementary material, which is available to authorised users.

## Introduction

The worldwide number of patients with diabetes is projected to increase to 440 million by 2030 [1]. One promising approach for the treatment of this disease is transplantation of insulin-producing cells derived from human pluripotent stem cells (hPSCs) [2–4]. Novel protocols have been described to derive functional beta cells in culture [5]. However, these protocols are complex and do not enable the expansion of pure beta cells. Most reports describe the derivation of mouse, monkey and human beta cells from stem cells, but only immature cells are produced and in low numbers [6–8]. In vitro hPSCs generate endocrine pancreatic precursors more readily than mature beta cells [6, 9]. Mature beta cells have been identified in vivo several weeks after murine transplantation of pancreatic progenitors that express pancreatic and duodenal homeobox 1 (PDX1) or homeobox protein Nkx-6.1 (NKX6.1) [9, 10]. hPSC-derived insulin-producing cells do not express the mature beta cell marker, urocortin-3 (UCN3), and have a low glucose threshold for insulin release [11, 12]. However, they acquire a higher, more physiological threshold and amplified insulin release upon glucose challenge after transplantation [12]. These facts strongly suggest that complex signals (the nature of which are currently unknown) may be essential to generate fully differentiated beta cells in vivo.

Endothelial cells (ECs) are major components of the pancreatic niche [13, 14]. ECs play a key role in organogenesis [8, 13, 15–18]; after induction from the notochord, pancreatic bud cells interact with ECs from the aorta and the dorsal vein to induce the formation of pancreas progenitors [13, 14, 19]. Aortic ECs are crucial for the survival of the pancreatic mesenchyme, including Islet-1^+^ cells, which signal to induce the expression of PDX1, a master regulator of pancreatic organogenesis [20]. The islet itself is one of the most highly vascularised structures in the body and components from the vascular basement membrane, as well as EC-derived factors, are essential for insulin gene expression in vitro and in vivo [19, 21–26]. Furthermore, the increase in islet microvasculature density coincides with the growth of islet endocrine cells observed after birth, suggesting a pivotal role for ECs in postnatal endocrine cell maturation [27]. Signals from ECs promote upregulation of bone morphogenetic protein 2 (BMP-2) and BMP-4 in mouse models, leading to the differentiation of several organs including the pancreas [8, 26]. Receptor activation of BMPs promotes mothers against decapentaplegic homologue (SMAD)-1, 5 and 8 phosphorylation and translocation to the nucleus, where they function as transcription factors [28]. Importantly, BMP-4 variants have been associated with a modest risk of type 2 diabetes mellitus, suggesting that BMPs may be fundamental to the integrity of beta cell function [29, 30]. In the present work, we used embryoid bodies (EBs), derived from reprogrammed or embryonic hPSCs, to investigate whether ECs enhance the in vitro generation of functional insulin-producing cells cultured in collagen–laminin scaffolds [21, 22, 31–33]. After labelling, isolating, expanding and characterising these cells, we tested their ability to release insulin in vitro, and also in vivo after transplantation under the kidney capsule of streptozotocin (STZ)-treated severe combined immunodeficient (SCID) mice [34, 35]. Additionally, we found consistent upregulation of BMP 2 and BMP4 only in EBs co-cultured with ECs, suggesting an important role for these peptides in the differentiation process.

## Methods

### Cells and reagents

The hPSC cell line, 83iCTR-n1, and the human embryonic stem cell line, H9 were obtained from the induced pluripotent stem cell (iPSC) core laboratory, Cedars-Sinai Regenerative Medicine Institute. These cells were negative for mycoplasma in the same facility before being used for differentiation experiments. At the core laboratory, hPSC derivation from human fibroblasts was performed according to published protocols [31, 32]. The hPSC line was used at passage 40–50; H9 cells were used at passage 20–30. Both cell lines were cultured in a feeder-free system with mTeSR1 basal medium plus 5× supplement (Invitrogen, Carlsbad, CA, USA), 200 μmol/l l-alanyl-l-glutamine (American Type Culture Collection [ATCC], Manassas, VA, USA) and 0.1 mmol/l β-mercaptoethanol (STEMCELL Technologies, Vancouver, BC, Canada). EBs were generated according to the manufacturer’s instructions and maintained in AggreWell medium (STEMCELL Technologies) supplemented with 10 μmol/l rho-associated protein kinase (ROCK) inhibitor (Sigma-Aldrich, St Louis, MO, USA). The human microvascular EC (HMEC) line was purchased from E. Ades and F. J. Candal (Centers for Disease Control and Prevention, Atlanta, GA, USA), and T. Lawley (Emory University, Atlanta, GA, USA). HMECs were negative for mycoplasma and were grown at 37°C under 5% CO_2_ and maintained in MCDB131 medium (Invitrogen) supplemented with 1% l-alanyl-glutamine (vol./vol.) (ATCC), 10% FBS (vol./vol.) (Omega Scientific, Tarzana, CA, USA) and 100 μg/ml endothelial cell growth supplement (Millipore, Hayward, CA, USA), for use at passages 20–25. For co-culture experiments, 100 EBs were picked up with a glass Pasteur pipette and 5 × 10^5^ HMECs inactivated with 100 μl/ml mitomicyn C (Sigma-Aldrich) were added to collagen I solution (BD Bioscience, Franklin Lakes, NJ, USA) with 1× minimum essential media (MEM), 1 mol/l 4-(2-hydroxyethyl)-1-piperazineethanesulfonic acid (HEPES) buffer, 7.5% bicarbonate solution (vol./vol.) (Life Technologies, Grand Island, NY, USA), 0.1 mmol/l NaOH, 1 mg/ml laminin I and 1 mg/ml collagen IV (R&D Systems, Minneapolis, MN, USA) and placed on ice. Then, 10 × 100 μl drops of cell suspension were placed into Petri dishes (60 × 20 mm; VWR, Cerritos, CA, USA) and solidified at 37°C for 10–20 min, after which, medium containing pancreatic differentiation factors including activin A, Wnt3a, retinoic acid (RA), fibroblast growth factor 7 (FGF7), epidermal growth factor (EGF), SB461542, hepatocyte growth factor (HGF), insulin-like growth factor-1 (IGF-1), and nicotinamide was added at different time points of EB development [7]. EB controls were cultured in gel without ECs but treated with the same pancreatic differentiation factors. After 20 days, cells were harvested using 5% collagenase I (wt/vol.) (Worthington, Lakewood, NJ, USA) and maintained in CMRL 1066 medium with CIT modification (Mediatech, Manassas, VA, USA), supplemented with 10 ml of 25% human serum albumin (HSA; NOVA Biologics, Oceanside, CA, USA) to a final concentration of 0.5% (vol./vol.), and 50 μl of a 1 mg/ml stock of IGF-1 (R&D Systems) at a final concentration of 10 ng/ml (vol./vol.). To generate definitive endoderm (DE) cells, undifferentiated iPSCs were cultured in a feeder-free system and culture medium, as described above. Subsequently, cells were transferred into six wells of a 24-well plate. At 90% confluency, RPMI 1640 (Life Technologies) containing 100 ng/ml activin A (Peprotech, Rocky Hill, NJ, USA) and 25 ng/ml Wnt3a (R&D Systems) was added to DE cells for the first day. On the second and third days, the cell medium was changed to RPMI 1640 containing only 100 ng/ml activin A. The DE cells were plated in collagen gel (as described above) with or without ECs, and pancreas differentiation factors were added [7]. The human hepatoma cell line and beta-TC-6 cells (ATCC) were both negative for mycoplasma.

### Immunocytochemistry

Cells were fixed with 4% paraformaldehyde (vol./vol.) (Polysciences, Warrington, PA, USA), permeabilised with 0.3% Triton X-100 (vol./vol.) in PBS and then incubated with primary antibodies, as outlined in the electronic supplementary material (ESM Table 1). The antibodies were validated by the providers and in our laboratory by staining frozen sections of human or mouse pancreases (positive controls) and hepatoma cell lines (negative controls) (data not shown). Images were acquired using a multipurpose zoom microscope (Nikon AZ 100; Nikon Instruments, Melville, NY, USA) attached to a DS-Qi1 high-sensitivity charge-coupled device (CCD) camera and analysed using NIS-Elements AR 3.10 (Nikon Instruments) and ImageJ 1.30v (National Institutes of Health, Bethesda, MD, USA) imaging software.

## FACS

Differentiated cells were harvested and stained with primary, secondary or isotype control antibodies (ESM Table 2). Cells were then analysed in a BD LSRFortessa cell analyser (BD Biosciences, San Jose, CA, USA) using fluorescence excitation at 360 nm and emission at 565 nm.

### Quantitative real-time reverse transcription-PCR analysis

Total RNA was isolated from cells using an RNeasy mini kit (Qiagen, Valencia, CA, USA). The QuantiTect reverse transcription kit (Qiagen) was used to synthesise cDNA and quantitative real-time reverse transcription PCR (qRT-PCR) analysis was performed using a SYBR Green RT-PCR kit (Qiagen) and a LightCycler instrument (Applied Biosystems, Foster City, CA, USA). PCR cycle conditions are shown in ESM Table 3. Analysis was performed using 7300 Sequence Detection Software (SDS) version 1.3 (Software Core Application, Applied Biosystems). Primer details are shown in ESM Table 4.

### Lentiviral plasmid construction and infection

A lentiviral reporter plasmid obtained from a viral core laboratory at Cedars-Sinai Regenerative Medicine Institute was constructed, containing a rat insulin minimal promoter that was amplified via PCR using the forward primer, 5′-CCCTCTAGACCGGCTGAGCTAAGAATCCAG-3′ (the XbaI sequence is underlined) and the reverse primer, 5′-GGCGACCGGTGCGGGAGTTACTGGGTCTCCACTAG-3′ (the AgeI sequence is underlined). After XbaI and AgeI restriction enzyme digestion, the PCR product was cloned into XbaI–AgeI sites upstream of the *mCherry* reporter gene in a self-inactivating second generation lentiviral vector containing a constitutively expressed hr*GFP-NLS* reporter under the control of the human ubiquitin C promoter. Following the differentiation protocol, 5 × 10^4^ insulin-producing cells/ml at passage 0 were plated into gelatin pre-coated 48-well plates. At 60% confluence, cells were transduced with the rat *INS-mCherry* lentiviral vector. Transduction efficiency was quantified as the number of cells expressing the *hrGFP-NLS* reporter.

### hPSC-derived beta-like cell isolation and expansion

After cell expansion, 1 × 10^6^ labelled cells at passage 1–2 were harvested to obtain a cell suspension for sorting using a BD FACSAria III cell sorter (BD Biosciences) with fluorescence excitation at 360 nm and emission at 565 nm to detect mCherry (red fluorescence signal) and green fluorescent protein (GFP; green fluorescence signal) expression. After sorting, cells were plated and passaged 3–7 times in CMRL 1066 media with CIT modification (Mediatech), supplemented with 10 ml of 25% HSA (wt/vol.) (NOVA Biologics) and 50 μl of 1 mg/ml IGF-1 (R&D Systems) for maintenance.

### Quinacrine secretion assay

Insulin-producing cells were plated onto 24-well plates at 1 × 10^5^ cells/ml and cultured at 37°C, 5% CO_2_ for 24 h. The complete medium was then replaced with medium containing 100 nmol/l quinacrine dihydrochloride (Sigma-Aldrich, St. Louis, MO, USA) and cells were incubated at 37°C, 5% CO_2_ for 30 min. Images were obtained by fluorescence microscopy at 360 nm excitation and 500 nm emission (Nikon AZ 100; Nikon Instruments). After washing with PBS, cells were incubated in RPMI 1640 supplemented with 0.1% BSA (wt/vol.) and different concentrations of glucose (0.5 mmol/l, 1.0 mmol/l, 2.8 mmol/l, 5.6 mmol/l or 16.5 mmol/l). Cell images were taken after 1 h with a DS-Qi1 high-sensitivity charge-coupled device (CCD) camera and analysed using ImageJ 1.30v (National Institutes of Health, MD, USA) software.

### Human insulin and C-peptide measurement

Human insulin and C-peptide were measured by ultrasensitive ELISA (Mercodia, Winston-Salem, NC, USA). The detection limits were 0.42 pmol/l for insulin and <2.5 pmol/l for C-peptide.

### Perifusion assay

Perifusion assays were performed using beta-TC-6 (positive control) or insulin-producing cells derived from co-cultures or controls [36]. Cells were perifused at 100 μl/min with Krebs-Ringer bicarbonate buffer (KRBH) containing 3 mmol/l glucose. After a 60 min equilibrium period (−60 to 0 min), cells were stimulated with 15 mmol/l glucose for 40 min, after which the perifusion solution was switched to 3 mmol/l glucose. Samples were collected every minute from −5 to 10 min; thereafter, samples were collected every 2 min from 12 to 26 min. Beta cell function was expressed as pmol/l insulin and as the percentage increase in insulin release relative to baseline.

### Cell transplantation in SCID mice

Animal experiments were approved by the Cedars-Sinai Animal Care and Use Committee. Thirty male SCID mice of the C.B-17/IcrHsd-*Prkdc* ^scid^ *Lyst* ^bg-J^ strain (Envigo, Indianapolis, IN, USA) were divided into three groups of ten mice. Animal care was undertaken by the Comparative Medicine department in agreement with the committee. The cages were housed in random order and physical randomisation was performed to assign mouse treatment (e.g. a piece of paper labelled with the animal ID number was withdrawn from a receptacle that was shaken and a treatment was assigned to this animal). All outcome assessments were carried out in random order by an investigator who was blind to the treatment groups. In the first group, 3 × 10^6^ insulin-producing cells derived from EBs co-cultured with ECs were transplanted under the kidney capsule. The second group received insulin-producing cells derived from EBs cultured alone. The third (control) group were injected with an equivalent volume of PBS. After 90 days, all mice underwent an IPGTT with 30% dextrose (wt/vol.) at 3 g/kg body weight. Blood samples were taken before glucose challenge and at 0, 30 and 60 min after injection. At 105 days after transplantation, mice were i.p. injected with STZ (Sigma-Aldrich) for 5 consecutive days (total dose: 350 mg/kg). A second IPGTT was performed 2 weeks after STZ treatment. Mice were euthanised approximately 126 days after transplantation (21 days after STZ treatment) and the left kidneys (harbouring the transplanted cells) were removed for immunohistochemical (IHC) analysis. The data from two animals from the control group and one animal transplanted with cells derived from EBs alone were excluded as the animals were euthanised due to moribund aspect after STZ injections.

## Immunohistochemistry

Insulin, glucagon and somatostatin levels were detected in kidney sections using a Leica DM 750 HD Digital Microscope (Leica Microsystems, Buffalo Grove, IL, USA) and commercially available primary antibodies (ESM Table 5) , after microwave heat-induced epitope retrieval using an automated detection system: either a Leica BOND-MAX (Leica Microsystems) or a Dako autostainer (Dako, Carpinteria, CA, USA).

### Statistical analysis

Data are expressed as the mean ± SE of three independent experiments. Student’s *t-*tests were used to identify significant differences between test groups (GraphPad Prism, La Jolla, CA, USA)

## Results

### ECs promote differentiation of hPSCs to insulin-producing cells

**Fig. 1:**
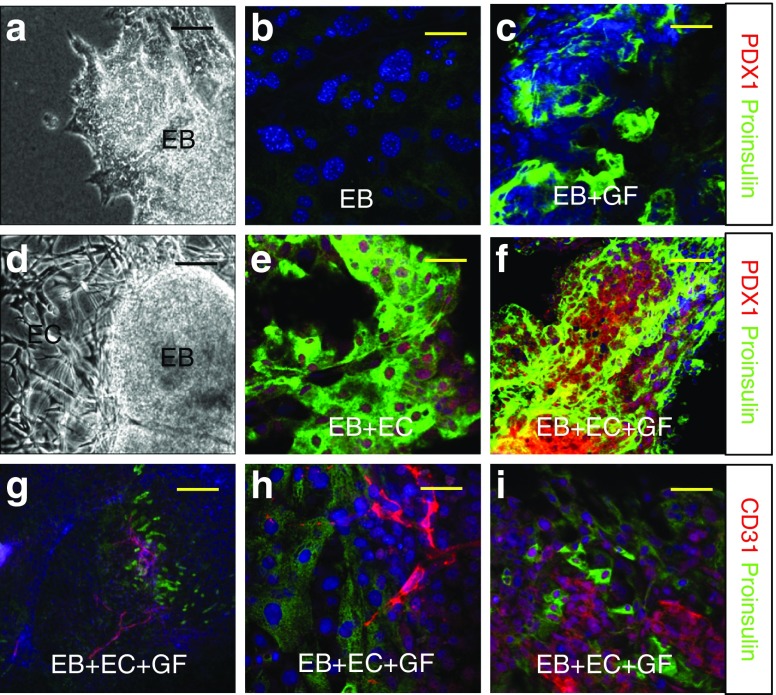
Co-expression of proinsulin plus PDX1 or proinsulin plus CD31 in EBs cultured alone or with ECs. EBs cultured (**a**–**c**) alone or (**d**–**f**) co-cultured with ECs were (**a**, **b**, **d**, **e**) untreated or (**c**, **f**) treated with growth factor (GF), then analysed by (**a**, **d**) bright-field light microscopy or co-stained for proinsulin (green) and PDX1 (red). (**g**–**i**) EBs co-cultured with ECs and treated with growth factors were co-stained with proinsulin (green) and CD31 (red); (**h**) confocal image. (**a**, **d**) Scale bar, 100 μm; (**b**, **c**, **e**, **f**, **i**) scale bar, 25 μm; (**g**) scale bar, 200 μm; (**h**) scale bar, 10 μm, *n =* 3

**Fig. 2:**
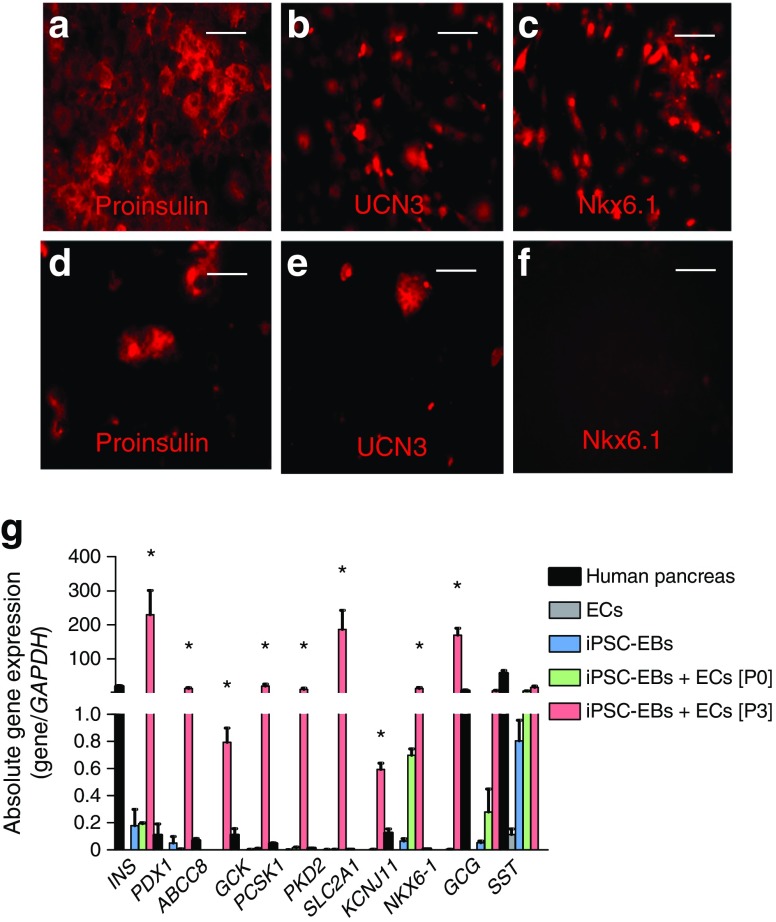
Analysis of pancreatic beta cell markers in EBs cultured alone or with ECs. (**a**–**c**) Co-cultured EBs or (**d**–**f**) EBs cultured alone were stained for (**a**, **d**) proinsulin (red), (**b**, **e**) UCN3 (red) or (**c**, **f**) Nkx6.1 (red). (**g**) Absolute gene expression in human pancreatic tissue, ECs, sorted cells derived from EBs cultured alone (iPSC-EBs), and sorted cells derived from EBs co-cultured with ECs at passage 0 (iPSC-EBs + ECs [P0]) and passage 3 (iPSC-EBs + ECs [P3]). *p < 0.05, *n =* 3

EBs cultured alone in collagen–laminin gels and not treated with growth factors were used as controls and compared with EBs co-cultured with ECs. EBs alone expressed lower levels of proinsulin and PDX1 (Fig. 1a–c). In contrast, co-cultured EBs (Fig. 1d) expressed a higher level of proinsulin and PDX1 (Fig. 1e) that was enhanced by the addition of growth factors (Fig. 1f). Tube-like structures were observed to surround co-cultured EBs (Fig. 1d) and a rich network of endogenous blood vessels expressing CD31 appeared contiguous with nascent insulin-producing cells (Fig. 1g–i). After harvesting, cells derived from co-cultures had increased expression of beta cell markers, such as proinsulin (Fig. 2a), UCN3 (Fig. 2b), and Nkx6.1 (Fig. 2c), compared with controls (Fig. 2d–f). Gene expression data corroborated the immunocytochemical observations, indicating a significantly higher level of beta cell marker expression in sorted cells derived from co-cultures at passage 3 (Fig. 2g) than in cells not co-cultured. Sorted cells also had significantly lower glucagon and somatostatin expression than insulin expression (Fig. 2g). FACS analysis showed that about 70% of cells from co-cultures were positive for proinsulin, in contrast to about 10% of growth factor-treated controls (ESM Fig.1a–f).

### Insulin-producing cells derived from EB–EC co-cultures can be labelled, isolated and expanded in vitro

**Fig. 3:**
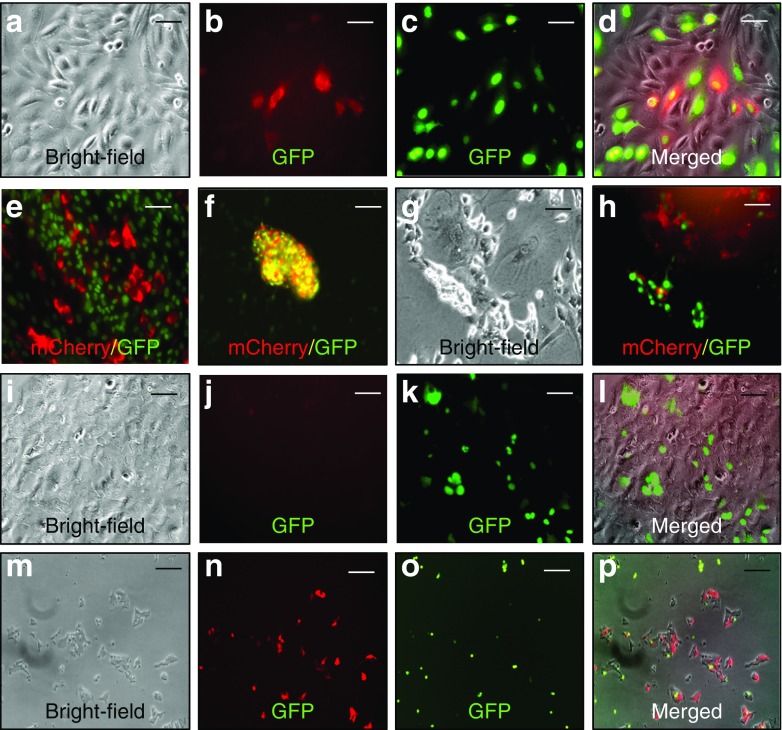
Isolation of insulin-producing cells from EBs cultured alone or with ECs. (**a**–**d**) Sorted insulin-producing cells derived from co-cultures were analysed by (**a**) bright-field light microscopy or labelled with (**b**) mCherry (red) or (**c**) GFP (green); (**d**) merged image. (**e**) Proliferation of insulin-producing cells. (**f**) Islet-like cluster formation. (**g**) Insulin-producing cells derived from EBs cultured alone and (**h**) mCherry and GFP expression in these cells. (**i–l**) Human hepatoma cells analysed by (**i**) bright-field light microscopy or labelled with (**j**) mCherry or (**k**) GFP; (**l**) merged image. (**m**–**p**) Beta-TC-6 analysed by (**m**) bright-field light microscopy or labelled with (**n**) mCherry or (**o**) GFP; (**p**) merged image. (**a**–**e**, **g**, **h**) Scale bar, 25 μm; (**f**, **i**–**p**) scale bar, 100 μm, *n* = 3

Labelled insulin-producing cells from co-cultured EBs expressed mCherry (Fig. 3a–d). These cells proliferated in vitro and formed islet-like clusters (Fig. 3e, f). Although they proliferated slowly, islet-like cluster formation became more frequent at later passages in cells plated in collagen-laminin gels (e.g. passage 3, as shown in ESM Fig 1g–j). In contrast, we observed very few viable cells expressing mCherry from EBs treated with growth factor in the absence of ECs (Fig. 3g, h). The human hepatoma cell line and beta-TC-6 cells were used as negative (Fig. 3i–l) or positive control (Fig. 3m–p) respectively.

### Insulin-producing cells derived from co-cultures respond efficiently to in vitro glucose challenge

**Fig. 4:**
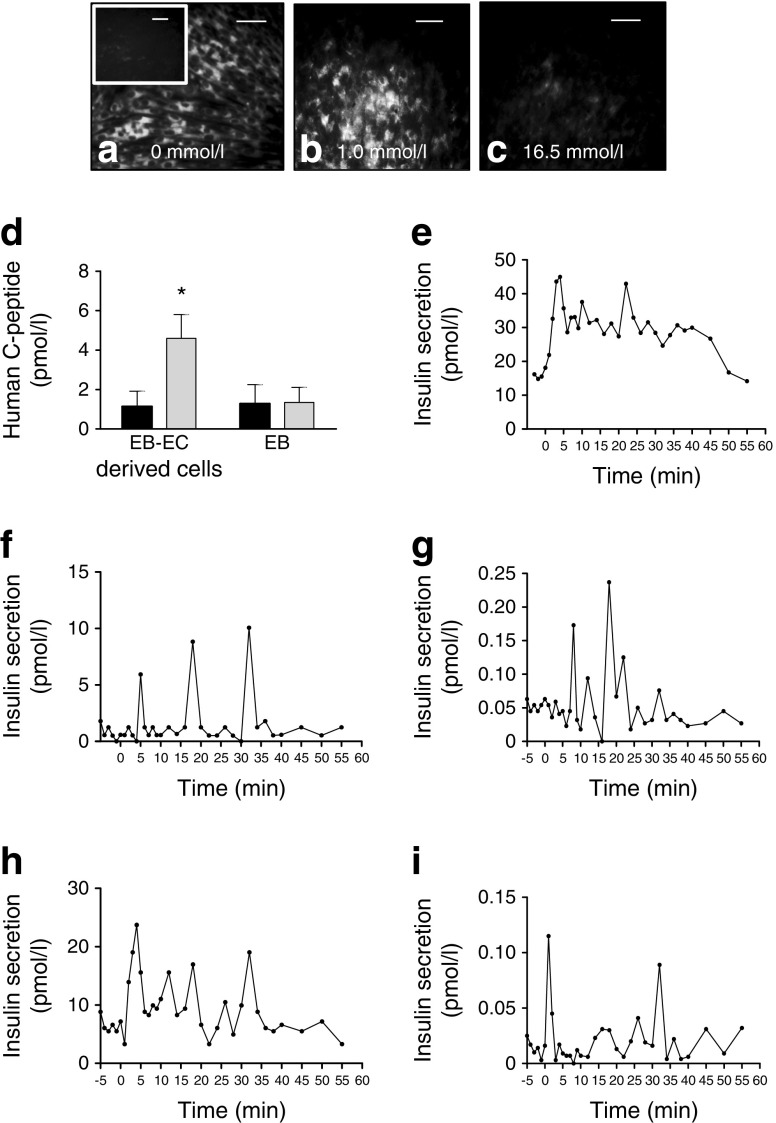
Quinacrine, human C-peptide and insulin secretion in insulin-producing cells derived from EBs cultured alone or with ECs. (**a**–**c**) Quinacrine was loaded for 30 min onto static cultures of co-cultured cells or (**a**) controls (inset), followed by the addition of (**b**) 1 mmol/l or (**c**) 16.5 mmol/l glucose. (**d**) Quantification of human C-peptide secretion in static cultures after 3 mmol/l (black bars) or 17 mmol/l (grey bars) glucose. (**e**–**i**) Dynamic insulin secretion (perifusion) was measured during 40 min after changing the glucose concentration from 3 to 15 mmol/l at time 0 and from 15 to 3 mmol/l at 40 min, in (**e**) beta-TC-6 cells, (**f**) hPSC-insulin-producing cells at passage 1, (**g**) hPSC-insulin-producing cells at passage 3, (**h**) H9-insulin-producing cells at passage 7 and (**i**) H9-insulin-producing cells at passage 15. (**a**–**c**) Scale bar, 25 μm; (**a**) inset scale bar, 25 μm *p < 0.05, *n* = 3

Quinacrine accumulates within insulin granules after 30 min; maximum fluorescence was detected in sorted cells derived from co-cultures (Fig. 4a), in contrast to low/no fluorescence in controls (Fig. 4a, inset). A significant decrease in fluorescence was observed after adding 1.0 mmol/l glucose and then increasing the concentration to 16.5 mmol/l (Fig. 4b, c). A threefold increase in human C-peptide levels was found in the medium from insulin-producing cells derived from co-cultures following challenge with 3 to 17 mmol/l glucose (Fig. 4d). In contrast, no C-peptide production was seen in EBs cultured alone (Fig. 4d). The kinetics of insulin secretion was quantified using a perifusion assay. Beta-TC-6 cells had an initial fast response followed by a slow response and oscillations (Fig. 4e). Human induced PSCs (hiPSCs) at passage 1 responded to glucose with an increasing amplitude in insulin oscillations that occurred with a frequency of about one oscillation every 12–14 min (Fig. 4f). At passage 3, the same cells showed more frequent oscillations with lower insulin peaks (Fig. 4g). Similarly, insulin-producing cells derived from the H9 human embryonic stem cell line exhibited a clear oscillatory response to glucose challenge, with higher insulin levels with more frequent oscillations at passage 7 (Fig. 4h) and lower insulin levels with less frequent oscillations at passage 15 (Fig. 4i).

### Pancreatic beta cells derived from EB–EC co-cultures respond to glucose challenge in vivo

**Fig. 5:**
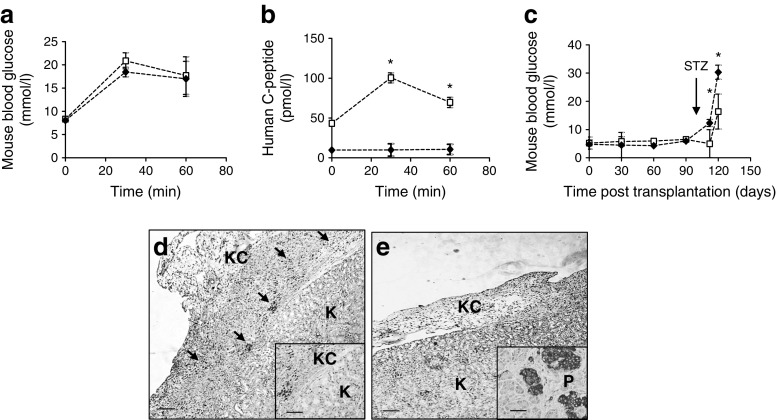
IPGTT and STZ treatment in mice grafted with insulin-producing cells derived from EBs cultured alone or co-cultured with ECs. (**a**) Blood glucose levels and (**b**) human C-peptide blood levels in mice transplanted with cells from EBs cultured alone (black squares) or EBs co-cultured with ECs (white squares). (**c**) Blood glucose levels in grafted mice before and after treatment with STZ. (**d**) Mouse kidney (K) and kidney capsule (KC) with grafted insulin-producing cells derived from EBs co-cultured with ECs that express insulin (arrows, insulin-positive cell clusters [stained black]). Inset shows the cells at higher magnification. (**e**) Images from control mice grafted with EBs cultured alone. Inset shows a normal human pancreas (P) with islets stained in black. Scale bars, 200 μm; (**d**) inset scale bar, 50 μm; (**e**) inset scale bar, 25 μm. *p < 0.05, *n* = 3

Sorted hPSC-derived insulin-producing cells, capable of responding to glucose in vitro, were expanded in culture. About 5 × 10^6^ cells at passage 3–4 were transplanted under the kidney capsule of SCID mice. Ninety days after transplantation, an IPGTT was performed. Following glucose challenge, the blood glucose level in control mice grafted with cells derived from EBs cultured alone ranged from 8 to 21 mmol/l with negligible human C-peptide detected (Fig. 5a, b). Concurrently, mice grafted with cells derived from co-cultures demonstrated nearly identical blood glucose levels but human C-peptide levels rose from 43 pmol/l at baseline to 100 pmol/l at 30 min and 69 pmol/l at 60 min (Fig. 5a, b). Mice were then treated with STZ to destroy native beta cells and glucose levels were measured 15 days later (Fig. 5c). Blood glucose levels were significantly lower in mice transplanted with co-cultured EBs (16.3 ± 6.1 mmol/l) compared with controls (30.3 ± 2.5 mmol/l, *p =* 0.0165) when assessed 2 weeks after STZ treatment. IHC analysis showed plentiful insulin-expressing cells in the kidney capsule of mice grafted with cells derived from co-cultures (Fig. 5d, black arrows); in contrast, there was no evidence of insulin expression in control mice (Fig. 5e).

## Discussion

During fetal development, a pancreatic beta cell mass results from islet neogenesis [36]. However, the factors involved in beta cell maturation in vivo are not completely understood. ECs are key components of the pancreatic niche and their absence can result in pancreatic agenesis [17, 21, 37]. In the present study, we used EBs and ECs to derive functional insulin-producing cells in vitro. We focused on promoting cell–cell interactions in vitro between ECs and human EBs, which are structures composed of cells derived from all three germ layers [38]. Our results indicate that insulin-producing cells are generated in vitro in EBs surrounded by ECs. These insulin-producing cells responded to glucose both in vitro and in vivo. Within the EBs, some ECs were located close to insulin-producing cells, suggesting that paracrine effects may also be essential to maintain the beta cell phenotype [8]. This hypothesis is supported by previous observations in mouse embryonic stem cells, where ECs promote beta cell maturation [8]. Beta cells also secrete soluble factors that affect the EC phenotype [39]. Islet ECs correspond to fenestrated endothelium [40]. Interestingly, beta cells exhibit polarity, with an apical and basolateral membrane and a greater density of insulin vesicles in the apical region close to ECs [14, 19]. These facts suggest that signals from beta cells and ECs are essential for beta cell function. For instance, insulin promotes an increase in nitric oxide synthase and vascular endothelial growth factor (VEGF)-A induces cell proliferation and maintains fenestrations in ECs [40]. In our experiments, these cell–cell interactions led to a higher yield and improved survival of insulin-producing cells and improved cellular function. We isolated, expanded and labelled these insulin-producing cells and found that the expression of mCherry (driven by the insulin promoter) decreased as the expression of GFP (driven by the ubiquitin C promoter) increased, suggesting that ubiquitination may interfere with insulin promoter activation, and thus limit the number of isolated insulin-producing cells [41–43]. Insulin-producing cells expressed markers of mature beta cells (UCN3, proinsulin, *INS*, *PDX1*, *NKX6-1*, *KCNJ11*, *ABCC8*, *GCK*, *PCSK1*, *PKD2* and *SLC2A1*). Some sorted cells also expressed islet cell markers (*GCG*, *SST*), suggesting that, following differentiation, the cell population included pancreas progenitors. In agreement with previous observations, insulin-producing cells from co-cultures proliferated in vitro [44]. However, decreased insulin expression resembling levels in islet cells was observed after in vitro expansion [45, 46]. Insulin-producing cells derived from co-cultures retained secretory ability, as assessed by quinacrine assay [47]. Perfusion assays and a threefold increase in C-peptide secretion after a glucose challenge in static cultures corroborated the beta cell phenotype in vitro. Additionally, we found oscillations in insulin secretion at higher glucose concentrations. This oscillatory secretion is essential for maintaining glucose homeostasis and a suitable insulin response in target cells [48]. Previous studies have also demonstrated an insulin secretion pattern in human beta cells derived from human embryonic stem cells [49]. Higher levels of C-peptide in vivo after glucose challenge and control of hyperglycaemia in STZ-treated mice strongly suggest that insulin-producing cells derived from the interactions between EBs and ECs maintain their functional capacity after transplantation in SCID mice. This finding could be relevant for the use of these cells as an alternative treatment for type 1 diabetes mellitus. In co-cultured EBs, we observed cell clusters that co-expressed p-SMAD1, 5 and 8, and a significant increase in BMP expression (ESM Fig. 2). Analysis of EC-conditioned medium showed that a higher level of BMP-2 and BMP4 expression was induced at stage three of differentiation in co-cultured EBs (ESM Fig. 3). Furthermore, the use of EC-conditioned medium improved the proliferation and survival of insulin-producing cells at various stages of differentiation (ESM Fig. 3). In contrast, less proliferation and more cell death was observed in EBs cultured alone. Thus, EC-derived factors in concert with pancreatic differentiation factors may potentiate cell survival, differentiation, proliferation and maturation in vitro. Finally, the effects of ECs were mimicked by adding a combination of BMP-2 and BMP-4 to DE cells that expressed CXCR4 and became insulin-producing cells (ESM Fig. 4).

In this study, we demonstrate that human dermal ECs provide essential signals for in vitro derivation of functional insulin-producing cells from hPSCs. These insulin-producing cells can be labelled, isolated and expanded, and maintain insulin secretion in vitro and in vivo. Optimised EB–EC interactions promote upregulation of BMPs that may be essential for the beta cell differentiation process. Long-term studies in animal models of diabetes are required to evaluate the capacity of these insulin-producing cells to maintain glucose homeostasis, for use in the treatment of type 1 diabetes mellitus.

## Electronic supplementary material

Below is the link to the electronic supplementary material.

ESM (PDF 666 kb)

## Abbreviations

BMP: Bone morphogenetic protein
DE: Definitive endoderm
EB: Embryoid bodies
EC: Endothelial cells
GFP: Green fluorescent protein
HMEC: Human microvascular endothelial cell
hPSC: Human pluripotent stem cell
IHC: Immunohistochemical
iPSC: Induced pluripotent stem cell
PDX1: Pancreatic and duodenal homeobox 1
SCID: Severe combined immunodeficiency
SMAD: Mothers against decapentaplegic homologue
STZ: Streptozotocin
UCN3: Urocortin-3
