# Supplementary material for: Effective endothelial cell and human pluripotent stem cell interactions generate functional insulin-producing beta cells
Source: Diabetologia. 2016 Aug 27;59(11):2378–86. doi: 10.1007/s00125-016-4078-1 (PMC5506104; doi:10.1007/s00125-016-4078-1)
Supplement: Supplementary file 1 — (PDF 666 kb) [file 125_2016_4078_MOESM1_ESM.pdf]

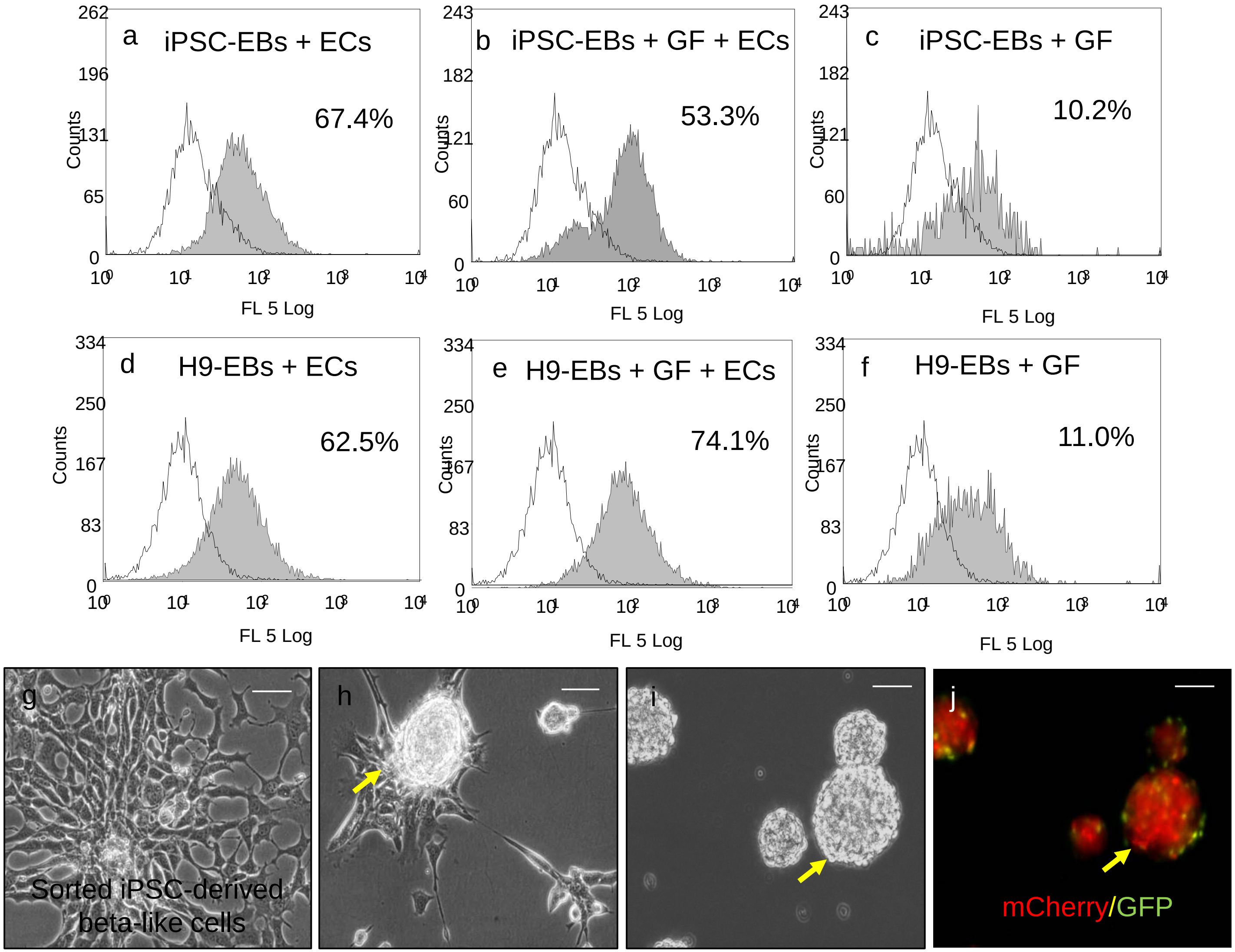

Fig. 1. FACS analysis and islet-like formation *in vitro* of hPSC-derived beta-like cells. Proinsulin expression evaluated by FACS of (a) hiPSC-derived EBs co-cultured with ECs. (b) hiPSC-derived EBs co-cultured with ECs and treated with GF. (c) hiPSC-derived EBs only treated with GF. (d) H9-derived EBs co-cultured with ECs. (e) H9 derived EBs co-cultured with ECs and treated with GF. (f) H9-derived EBs only treated with GF. (g) Bright field of sorted beta-like cells derived from co-cultures plated on collagen pre-coated dishes at P3 after sorting. (h) A different field showing cells that form a cluster (yellow arrow). (i) Islet-like structure (yellow arrow). (j) Islet-like cluster that expressed mCherry (driven by insulin promoter) and (GFP) driven by ubiquitin C promoter. Bar = 25 µm. *n* = 3.

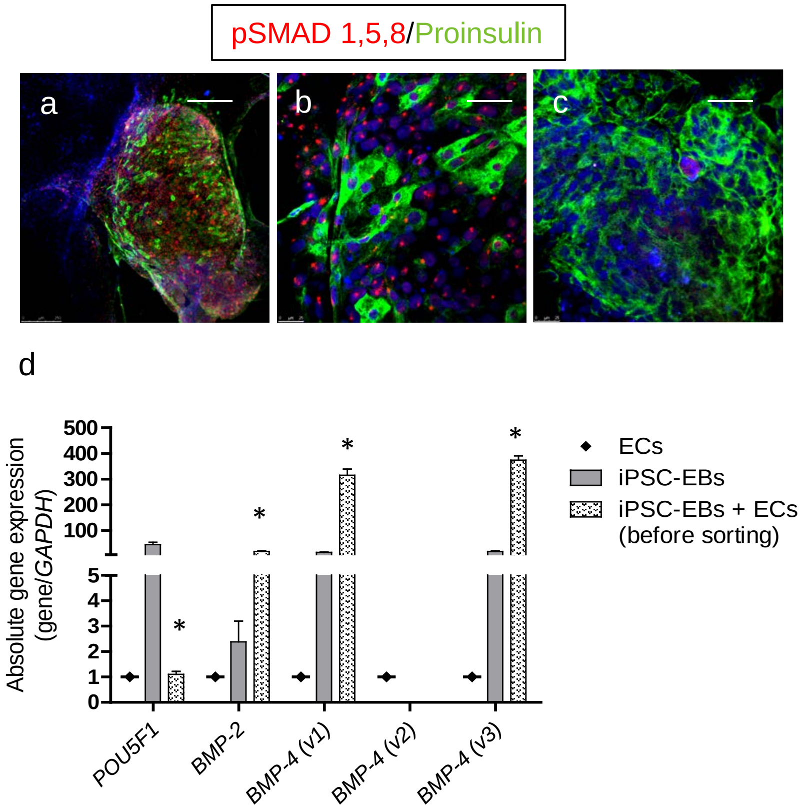

Fig. 2. Analysis of proinsulin expression, BMP-2/-4 upregulation and SMAD1,5,8 phosphorylation in EBs cultured alone or co-cultured with ECs. (a) Lower magnification of a cluster composed of cells positive to proinsulin (green) and pSMAD1,5,8 (red) found in EBs co-cultured with ECs. (b) Higher magnification of the same cluster. (c) Higher magnification of a cluster found in EBs cultured alone. (d) Expression of POU5F1, BMP-2, BMP-4 variant 1 (v1), BMP-4 variant 2 (v2), and BMP-4 variant 3 in human microvascular endothelial cells (HMECs), non-sorted cells from EBs cultured alone (iPSC-EBs) or non-sorted cells from EBs co-cultured with ECs. * $P < 0.05$, $n=3$.

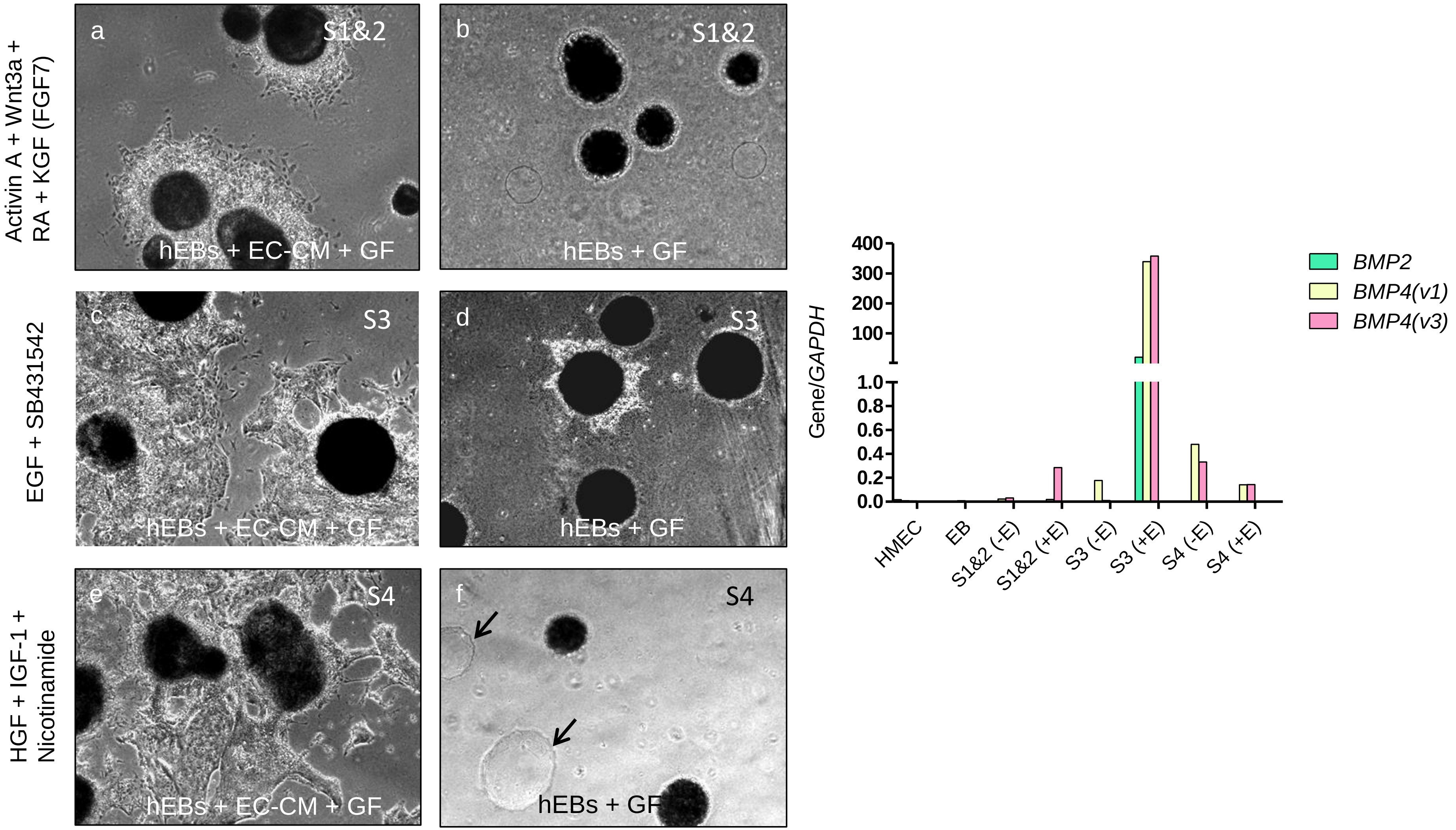

Fig. 3. Morphology of human iPSC-derived EBs treated or untreated with EC-conditioned medium (EC-CM) and evaluation of BMP-2/-4 expression. All EBs were treated with pancreatic differentiation factors (GF) and plated in collagen-laminin gels for 20 days. Some EBs were also treated with EC-CM during the four differentiation steps (S1 [activin A, Wnt3a], S2 [retinoic acid and fibroblast growth factor-7], S3 [epidermal growth factor and SB431542], and S4 [hepatocyte growth factor, insulin-like growth factor-1, exendin-4, and nicotinamide]). Human iPSC-derived EBs at S1 and S2 treated (a) or untreated (b) with EC-CM. Human iPSC-derived EBs at S3 treated (c) or untreated (d) with EC-CM. Human iPSC-derived EBs at S4 treated (e) or untreated (f) with EC-CM. (g) Quantification of BMP-2 or two variants of BMP-4 (v1, v3) in EBs at different stages of differentiation in EBs treated with GF in presence (+E) or absence (-E) of ECs. Black arrows indicate empty spaces were EBs detached. Bar = 100 µm, *n=3*.

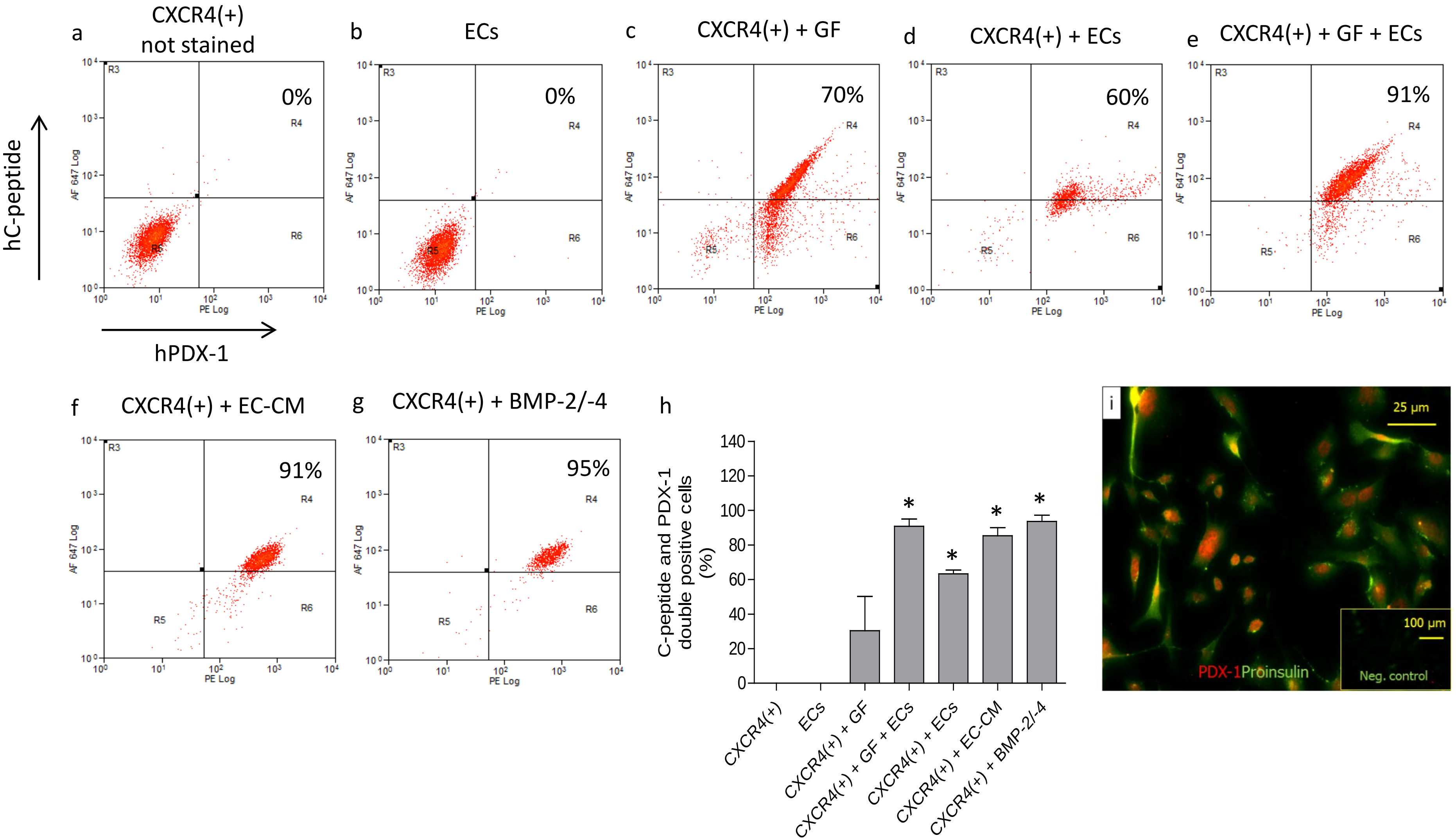

Fig. 4. Effects of EC-conditioned medium (EC-CM) or BMP-2/-4 in the differentiation of definitive endoderm (DE) to insulin-producing cells. Co-expression of proinsulin and PDX-1 evaluated by FACS in (a) non-stained definitive endoderm (DE; CXCR4+) derived insulin-producing cells, (b) ECs, (c) DE cells treated with growth factors (GF), (d) DE cells co-cultured with ECs (no treated with GF), (e) DE cells co-cultured with ECs and treated with GF, (f) DE cells treated with GF and EC-CM, (g) DE treated only with a combination of BMP-2 and BMP-4 (100 ng/ml each). (h) Quantification of FACS results. * $P < 0.05$. (i) Co-expression of proinsulin and PDX-1 in beta-like cells after treatment of DE cells with EC-CM (use as an example) evaluated by ICC. Inset shows cells stained with isotype antibodies. Bar = 25 μm. Bar Inset = 100 μm, *n*=3.

Table 1. Primary and secondary antibodies used for immunocytochemistry.

Antibody	Dilution/Concentration	Company
Proinsulin	1:100	Millipore, Billerica, MA, USA
Phospho-SMAD1,5,8	1:100	Cell Signaling, Danvers, MA, USA
PDX-1	1:100	Abcam, San Francisco, CA, USA
CD31	1:10	Abcam, San Francisco, CA, USA
UCN3	5 µg/ml	Abcam, San Francisco, CA, USA
Nkx6.1	1:100	Developed by Ole D. Madsen and obtained from the Developmental Studies Hybridoma Bank developed under the auspices of NICHD and maintained by the University of Iowa, Iowa City, IA 52242, USA
Normal mouse IgG_1	1:100	Santa Cruz Biotechnology, Inc., Santa Cruz, CA, USA
Normal rabbit IgG	1:50	Santa Cruz Biotechnology, Inc., Santa Cruz, CA, USA
Alexa Fluor 555 goat anti-rabbit IgG	1:1000	Molecular Probes, Eugene, OR, USA
Alexa Fluor 555 goat anti-mouse IgG	1:1000	Molecular Probes, Eugene, OR, USA

Table 2. Primary and secondary antibodies used for FACS analysis.

Antibody	Dilution/Concentration	Company
Proinsulin	1:100	Millipore, Billerica, MA, USA
PDX-1	1:50	Abcam, San Francisco, CA, USA
Alexa 488 anti-mouse IgG	1:1000	Life Technologies, Gran Island, NY, USA
Alexa 555 anti-goat, and Alexa 555 anti-rabbit	1:1000	Life Technologies, Gran Island, NY, USA
Normal rabbit IgG	1:100	Santa Cruz Biotechnology, Inc., Santa Cruz, CA, USA
Normal mouse IgG_1	1:100	Santa Cruz Biotechnology, Inc., Santa Cruz, CA, USA

Table 3. Protocol for qPCR

Step	Step 1	Step 2
Condition	95ºC, 10 min	94ºC, 30 sec; 60ºC, 20 sec; 72ºC, 30 sec
Cycle	1	45

Table 4. Forward and reverse primers (sequences are 5´-3´).

Gene	Primer Forward	Primer Reverse
GAPDH	AGCCACATCGCTCAGACACC	GTACTCAGCGGCCAGCATCG
INS	AGCCTTTGTGAACCAACACC	GCTGGTAGAGGGAGCAGATG
PDX-1	GGATGAAGTCTACCAAAGCTCACGC	CCAGATCTTGATGTGTTCTCTCGGTC
SUR1	GTGCACATCCACCACAGCACATGGCTTC	GTGTCTTGAAGAAGATGTATCTCCTCAC
GKS	AAGAAGGTGATGAGACGGATGC	CATCTGGTGTTTGGTCTTCACG
PC1/3	TTGGCTGAAAGAGAACGGGATACATCT	ACTTCTTTGGTGATTGCTTTGGCGGTG
PC2	GCATCAAGCACAGACCTACACTCG	GAGACACAACCACCCTTCATCCTTC
GLUT1	TCACTGTGCTCCTGGTTCTG	CTTGTGCTCCTGAGAGATCC
Kir6.2	CGCTGGTGGACCTCAAGTGGC	CCTCGGGGCTGGTGGTCTTGCG
Nkx6.1	CTGGCCTGTACCCCTCATCA	CTTCCCGTCTTTGTCCAACAA
GCG	AGGCAGACCCACTCAGTGA	AACAATGGCGACCTCTTCTG
STT	GTACTTCTTGGCAGAGCTGCTG	CAGAAGAAATTCTTGCAGCCAG
OCT4	CGACCATCTGCCGCTTTG	GCCGCAGCTTACACACATGTTCT
BMP-2	AAAACGTCAAGCACCACACAAA	GTCACTGAAGTCCACGTACAAAGG
BMP-4 (v1)	CCAAGCGTAGCCCTAAGCAT	GGCGCCGGCAGTTCTT
BMP-4 (v2)	GCCAAGCGTAGCCCTAAGC	CGCCGGCAGTTCTTATTCTT
BMP-4 (v3)	GCCAAGCGTAGCCCTAAGC	CGCCGGCAGTTCTTATTCTT

Table 5. Primary antibodies used for immunohistochemical analysis.

Antibody	Dilution/Concentration	Company
Insulin	1:500	Dako, Carpinteria, CA, USA
Somatostatin	1:500	Abcam, San Francisco, CA, USA
Glucagon	1:500	Abcam, San Francisco, CA, USA
